# The benefits, barriers, and specific needs of palliative care for adults with cancer in sub-Saharan Africa: a systematic review

**DOI:** 10.1080/16549716.2025.2485742

**Published:** 2025-04-10

**Authors:** Fawziyyah Usman Sadiq, Yu-Lyu Yeh, Hung-En Liao, Muhammad Alwi Eka Pranata, Sneha Patnaik, Yin-Hwa Shih

**Affiliations:** a Department of Healthcare Administration, Asia University, Taichung, Taiwan; b Department of Biology, Microbiology and Science Laboratory Technology, Nile University of Nigeria, Abuja, Nigeria; c Public Health Department, Universitas Muhammadiyah Kalimantan Timur, Samarinda, Indonesia

**Keywords:** Supportive care, adult patients, oncology, low resource settings, quality of life

## Abstract

People living in low – and middle-income countries are less likely to receive palliative care. Early delivery of palliative care reduces unnecessary hospital admissions and improves patients’ and their families’ quality of life. This systematic review has compiled and scrutinized adult cancer patients’ benefits, barriers, and specific palliative care needs in sub-Saharan Africa (SSA) to provide stakeholders with vital information that can improve the support and care provided to this expanding patient population. A systematic literature search was conducted using PubMed, Cumulative Index of Nursing and Allied Health Literature (CINAHL), Embase, Medline, and ProQuest under the Preferred Reporting Items for Systematic Reviews and Meta-Analyses (PRISMA) guidelines. Studies included in the review focused on the utilization of palliative care among adult cancer patients in sub-Saharan Africa. The Joanna Briggs Institute (JBI) Critical Appraisal tools assessed study quality. This review synthesized findings from 16 studies and highlights that access to palliative care improves cancer patients’ quality of life, satisfaction, and outlook on diagnosis. However, limited knowledge, financial constraints, and systemic obstacles impede access. Patients’ needs were categorized into four themes: physical comfort, psychosocial support, spiritual well-being, and socioeconomic assistance. Palliative care improves adult cancer patients’ quality of life in sub-Saharan Africa, but barriers hinder access. To address the challenges and meet patients’ needs, enhancing literacy about palliative care, providing financial support, and implementing structured and sustainable palliative care models are essential for strengthening services and improving regional healthcare.

## PAPER CONTEXT

- **Main findings**: Enhancing literacy about palliative care, providing financial support and implementing structured and sustainable palliative care models are essential for strengthening palliative care services and improving regional healthcare.
- **Added knowledge**: Our review explores the potential of adopting Swedish palliative care guides, which have positively impacted patient outcomes in palliative care, to strengthen palliative care in sub-Saharan Africa, where such frameworks have not yet been widely implemented.
- **Global health impact on policy and action**: This paper advocates for bold global health policy actions to enhance palliative care for cancer patients, particularly in sub-Saharan Africa, through raising awareness, securing funding, integrating care into national cancer strategies, and fostering multi-stakeholder collaboration to overcome barriers, address unmet needs, and promote sustainable, equitable health outcomes globally

## Background

The need for palliative care cannot be overemphasized, especially in an aging society and in an era where millions of people are diagnosed with non-communicable diseases (NCDs) and life-threatening illnesses yearly. Palliative care is a holistic and essential component of healthcare, which employs a team-based approach to provide relief from the symptoms, pain, and psychological distress associated with serious illness. It plays a vital role in improving the quality of life for patients through managing symptoms, offering psychological support, and addressing spiritual needs [1]. However, despite its importance, access to palliative care remains a significant challenge, particularly in sub-Saharan Africa (SSA), where health-care systems are often under-resourced and strained by the rising burden of NCDs, such as cancer [1,2].

Globally, cancer is a leading cause of morbidity and mortality, with incidence rates particularly rising in developing countries [3]. In SSA, cancer is among the top three causes of premature death, contributing to a significant portion of the 1.3 million new cancer cases and 800,000 cancer-related deaths reported in the region annually. Common malignancies affecting adults in SSA include breast cancer, cervical cancer, prostate cancer, gastrointestinal cancers (such as colorectal and liver cancer), and hematologic cancers [4]. Estimates suggest that by 2040, the cancer burden in SSA will nearly double, with 1.5 million new cases and over 1 million deaths, driven by factors, such as population growth, aging, and increasing adoption of risk behaviors [5].

As populations age, cancer susceptibility increases due to declining cellular repair mechanisms and accumulation of risk factors over time, making cancer a persistent health challenge, especially in resource-limited settings [6]. In particular, advanced-stage cancers, such as gynecologic cancers, sarcomas, lung cancer, and nasopharyngeal cancer, are frequently diagnosed due to late presentation and limited screening programs in SSA. This burden further underscores the need for effective cancer care and palliative services, especially for the aging adult population in SSA, who are vulnerable and face unique barriers to accessing adequate healthcare [3,7].

Given the projected increase in cancer cases and the aging population in SSA, it is imperative to scale up palliative care services in the region [1]. Therefore, this systematic review aims to assess palliative care access for adults with cancer in SSA, focusing on service availability, benefits, barriers, patient expectations, and strategies for improving care delivery. By identifying challenges and opportunities for improvement, this review seeks to strengthen palliative care efforts and, ultimately, enhance the quality of life for cancer patients across the region.

## Methods

The systematic review used the Preferred Reporting Items for Systematic Reviews and Meta-Analyses (PRISMA) guidelines, 2020 iteration [8]. To ensure transparency and adherence to best practices in reporting, the protocol for this review was registered with the PROSPERO International Prospective Register of Systematic Reviews, bearing the registration number CRD42023492496. Using this approach, the review aimed to comprehensively explore access to palliative care for adult cancer patients living in SSA.

### Search strategy and selection

A comprehensive literature search was conducted across five databases: PubMed, Cumulative Index of Nursing and Allied Health Literature (CINAHL), Embase, Medline, and ProQuest. Citation searching was also performed by reviewing the reference lists of key articles and relevant reviews. This led to identifying seven additional studies not captured in the initial database search. The search was based on a set of key terms: (‘Adults’ OR ‘older adults’ OR ‘elderly’ OR ‘geriatrics’ OR ‘senior citizens’ OR ‘aged’) AND (‘Cancer’ OR ‘Tumour’ OR ‘Neoplasm’ OR ‘Carcinoma’ OR ‘Malignancy’) AND (‘Healthcare’ OR ‘Health maintenance’ OR ‘Medical management’ OR ‘Preventive medicine’ OR ‘Wellness program’ OR ‘Palliative care’ OR ‘Hospice care’ OR ‘End-of-life care’) AND (‘Sub-Saharan Africa’ OR [list of countries]). The search was restricted to studies published between 2013 and September 2023 to focus on recent findings, as palliative care in the region was in its early stages before 2013. For this review, ‘adults’ are defined as individuals aged 18 and above, while ‘older adults’ refers to individuals aged 65 and above. Initially, the focus was on older adults. Still, due to the limited research explicitly targeting this group in SSA, studies involving all adult age groups were included to ensure a comprehensive analysis.

### Inclusion and exclusion criteria

This systematic review included studies that met the following criteria: 1) studies that focused on the experiences of adult cancer patients in SSA, 2) studies that examined factors that influenced the use of palliative care among adult cancer patients in SSA, 3) studies that were published in peer-reviewed journals, 4) studies that were written in English, and 5) studies with full-text availability. The review included original research without restrictions on research design, allowing for a comprehensive inclusion of relevant findings. Both quantitative, qualitative, and mixed-methods studies were eligible for inclusion. Studies were excluded if they: 1) focused on diseases other than cancer, 2) did not focus on adults (aged 18 and above), 3) did not address the utilization as well as factors associated with access to palliative care, 4) were editorial, commentaries, case studies, or research protocols. 5) were systematic review studies.

### Data screening and extraction

**Figure 1.**
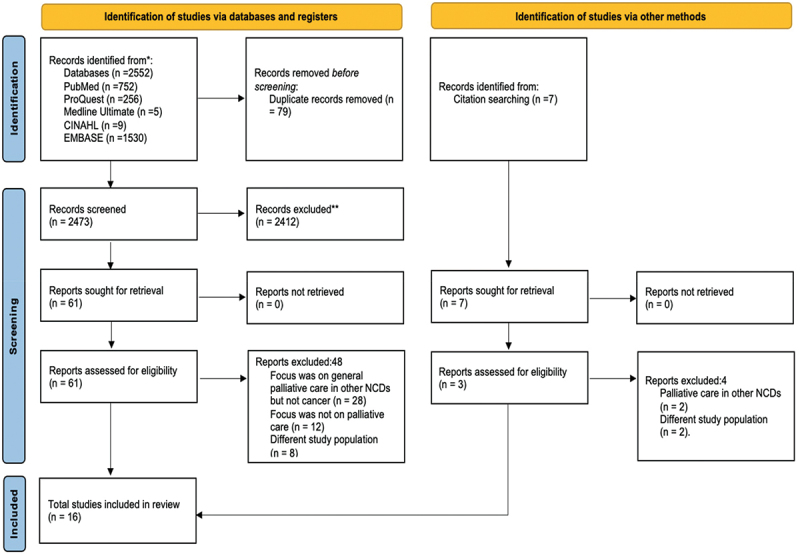
PRISMA flow diagram illustrating the systematic literature search and selection process.

Duplicate references were systematically identified and removed before the screening and data extraction. The article selection process is illustrated in Figure 1. Two reviewers (F.S. and M.A.) independently reviewed the titles and abstracts of each article to determine eligibility. Full texts of potentially eligible articles were retrieved for further review. The reviewers then independently screened the full texts and assessed each article’s eligibility through discussion and consensus. In cases of disagreement, the two reviewers reconciled the decision, and a third reviewer (S.P.) was consulted as needed until an agreement was reached. This process resulted in the selection of 16 final articles. Data from these 16 eligible articles were extracted independently by the two reviewers (F.S. and M.A). The extracted data included authors, year, study location, study design, participant demographics (sex and age range), type of cancer, mode of palliative care delivery, and reported results (benefits, challenges, and expectations). The extracted data were recorded in a Microsoft Excel 2010 spreadsheet. The reviewers met regularly to analyze, discuss, and resolve discrepancies during data extraction.

### Quality appraisal

The quality of the included studies was assessed using the Joanna Briggs Institute (JBI) [9] checklist for critical appraisal. Two independent reviewers evaluated the evidence level of each article. Any disagreements were resolved through consensus discussions. The agreement between the two reviewers for the title and abstract screening was substantial (Kappa = 0.72, *p* < 0.001). Similarly, the agreement for full-text screening and quality assessment was also substantial (Kappa = 0.76, *p* < 0.001 and Kappa = 0.87, *p* < 0.001, respectively). All selected studies met the quality assessment criteria.

### Data synthesis

Thematic analysis was conducted on the included studies following established synthesis guidelines [10]. This process involved identifying key themes, comparing and refining concepts, and organizing the patterns and relationships between the various themes and keywords. The analysis was grounded in the initial descriptive themes generated, allowing a deeper understanding of the data.

## Results

### Study selection

The initial search across five databases identified 2,559 articles, including seven manually sourced. After removing 79 duplicates, 2,480 articles were screened by title and abstract, resulting in 2,412 being excluded. The full texts of the remaining 68 articles were reviewed, and 52 were further excluded. Ultimately, 16 studies met the inclusion criteria and were included in the synthesis. The detailed review process is shown in Figure 1.

### Study description and characteristics

Among the 16 studies reviewed, eight were quantitative, seven were qualitative studies, and one was a mixed-method study. The majority of studies were cross-sectional, with only three using retrospective cohort data. Studies were conducted across various SSA countries, with South Africa, Nigeria, and Ethiopia represented the most frequently. Regarding cancer types, many studies have focused on female cancers, particularly breast and cervical cancer. In contrast, others involved a broader range of cancers affecting both males and females, including prostate, gastrointestinal, and hematologic cancers. Detailed study characteristics are presented in Tables 1 and 2.

**Table 1.** A summary of the description of selected quantitative studies, participants’ demographics and characteristics of palliative care.

Authors Year Country Article no.	Study Design	Participants	Age	Type of Cancer	Standardized scale	Palliative Care Characteristics
Akuoko et al. 2022 Ghana [19]	Cross-sectional study	176 females	25–82	Advanced breast cancer	Supportive care needs survey-long form, the Spiritual Need Assessment for Patients and the modified Client Service Receipt Inventory	Hospital outpatient services (public and private health institutions)
Lazenby et al. 2016 Botswana [20]	Cross-sectional study	65 females; 35 males	22–83	Gynaecological, sarcomas, breast, hematologic, gastrointestinal cancers	Memorial symptom assessment scale-short form (MSAS-SF), Enforced social dependency scale (ESDS)	Oncology wards of [1] a public hospital [2], a private hospital and [3] a hospice (operated by a non-governmental organisation).
Omoyeni, et al. 2014 Nigeria [12]	Cohort study	20 males; 40 females	19–98	Breast, prostate, cervical, gastrointestinal, and liver cancers	Pain was assessed using the Numerical Rating Scale (NRS): 0 = No pain,10 = Worst possible pain	Home-based palliative care. Homes were visited 1–23 times per person. Services offered were symptom control, psychosocial counselling, carer training, physiotherapy services, financial and bereavement support, as well as other support (comfort packs consisting of food and toiletries) provided by a not-for-profit organisation and individual donations
Olaitan et al. 2016 Nigeria [16]	Cohort study	44 males; 77 females	21–91	Various cancer cases (11 types)	Retrospective records	Outpatient and home-based palliative service within 5–224 days providing pain and symptom control, counselling, education for patients and family, as well as financial and spiritual support provided by a university college hospital in collaboration with an NGO
Tarus et al. 2022 Kenya [24]	Cohort study	62 females	23–86	Breast cancer	Retrospective chart and medical records	Palliative care obtained from inpatient hospice. Pain and symptom management, psychosocial support as well as end-of-life care was given to patients
Harding et al. 2014 Kenya and Uganda [21]	Cross-sectional study	126 females; 84 males	18–86	Advanced cancer cases	Self-report data, African Palliative outcome scale (POS)	Uganda (Hospice in the capital city which provides home and daycare) Kenya (1^st^ site: hospice in the capital city Nairobi, which cares for patients at different points, i.e. at the hospice, home visits, hospital visits, and a mobile clinic in one of the largest informal settlements within the city environs and 2^nd^ site: is a rural hospice that provides holistic care within the hospice, day care services, hospital consultations as well as holding a monthly legal aid clinic)
Amare et al. 2023 Ethiopia [17]	Cross-sectional study	176 females; 125	20–71	Gynaecological, breast and nasopharyngeal cancers (top 3 cancers)	The palliative care indicators tool in low-income settings (SPICT-LIS)	Hospital-based palliative care (Details of care not mentioned)
Ndiok et al. 2018 South Africa [15]	Cross-sectional design	547 participants	18–90	All cancer types/patients	Survey questionnaire	Hospital-based care from two tertiary health institutions
Dlamini et al. 2020 South Africa [13]	Cross-sectional study (Mixed method study)	306 females, 84 males	30–70	Cervical, breast and other types of cancers (Kaposi’s sarcoma, prostate, colon, anal, vulva & lung cancer).	Structured questionnaire; two open -ended questions	Hospital-based care from five different hospitals in the study area

**Table 2.** A summary of the description of selected qualitative studies, participants’ demographics and characteristics of palliative care.

Authors Year Country Article no.	Study Design	Participants	Age	Type of Cancer	Data Collection method	Palliative Care Characteristics
Kebebew et al. 2022 Ethiopia [18]	Cross-sectional study	385 females	20–80	Advanced Cervical cancer	Face to face interviews using structured questions	Tertiary hospital-based palliative care (consisting of physical, psychological, and mental healthcare and social and economic support)
Lambert et al. 2020 South Africa [14]	Cross-sectional study	50 females	28–76	Breast cancer	In-depth interviews	Hospital care (public hospital sometimes, NGO) and familial/social support (via family, loved ones, friends, and support groups offering religious/faith support and counselling
Natuhwera et al. 2021 Southwestern Uganda [25]	Cross-sectional study	10 females	40–71	Cervical cancer	Semi-structured interview	Mobile hospice (a branch of an NGO) offering counselling, religious support, medication for pain and symptom control practical support and welfare funding for transport and drugs)
Nkoana et al. 2022 South Africa [26]	Cross-sectional study	20 males	67–85	Prostate cancer	In-depth semi-structured individual interview	Coping Support through family, healthcare providers and, majorly, religion
Namukwaya et al. 2022 Uganda, Nigeria and Zimbabwe [23]	Cross-sectional study	27 males; 35 females	18 and above (Mean age 51.61)	Advanced cancer cases (18 types)	In-depth interviews	Community-based palliative care was primarily available in Uganda and Zimbabwe, where home visits by care teams were primarily made. Review after chemotherapy by participants from Nigeria. Phone-based palliative care service
Chona et al. 2023 Tanzania [22]	Cross-sectional study	12 females	31–90	Cervical cancer	Semi-structured face-face interview	Palliative care in ORCI (cancer institute), a national referral public centre for cancer treatment, provides supportive and palliative care
Anarado, et al. 2017 Nigeria [11]	Cross-sectional design	20 females	36–66	Breast cancer	Focus group discussion	Outpatient hospital department and care support were administered by nurses

### Participants demographics

The study population consisted of adults with cancer from sub-Saharan Africa (SSA), with sample sizes ranging from 10 to 547 participants aged between 18 and 98. The study by Anarado et al. [11] had the narrowest age range (36–66), while Omoyeni et al. [12] had the widest range (19–98). Also, female participants outnumbered male participants. A summary of the demographic characteristics is provided in Tables 1 and 2.

### Palliative care services in included studies

Of the 16 studies reviewed, 10 [11,13–21] reported that participants received hospital-based palliative care, with three [11,12,19] specifying outpatient services. Two studies [19,20] indicated that public and private health institutions provide these services, while one [14] mentioned a public hospital. One study [22] highlighted care at a national referral public center for cancer treatment. Additionally, four studies [12,16,21,23] described home-based palliative care, with some patients receiving home visit classes [21] and phone-based palliative care [23].

Four studies [20,21,24,25] also mentioned hospice care, including mobile [25] and inpatient hospice services [24]. Some palliative care services are supported by NGOs [12,14,16,20,25], and one study noted individual donations as a source of support [12]. Alongside medical care, patients received coping and supportive care from friends and family [14,26], religious groups [14,26], and healthcare providers [11,26]. The palliative care provided covered a range of needs, including physical [12,16,18,24], psychological [12,18,24], social [12,14,18,24], economic/financial support [12,16,18], mental health [18], and religious support [16,25,26]. Tables 1 and 2 also show the palliative care support received by patients.

#### Benefits of access to palliative care

The benefits of access to palliative care, as identified in 11 reviewed studies, were categorized into three main areas: improved quality of life, patient satisfaction, and a better attitude towards diagnosis.

##### Improved quality of life

Out of the selected studies, nine [12,14,16,18,21,23–26] indicated that palliative care led to improved quality of life, with benefits including reduced suffering and pain relief [12,16,24] access to anti-pain treatments [18] improved survival rate through affordable healthcare [14] enhanced spiritual and psychological well-being [21,26] better relationships [25] quicker service response and improved symptom management [23].

##### Patient satisfaction

Five selected studies [12,14,23,25,26] highlighted that patients’ felt supported, cared for, and relieved through palliative care, resulting in greater satisfaction. This is a result of the reduced hospital visits and appropriate treatment plans [12], family support and financial assistance [14,25,26] as well as better symptom management and prompt care, which also inspired hope for a cure [23].

##### Better attitude towards diagnosis

Five studies [11,14,21,25,26] also indicated that palliative care helped patients develop a more positive attitude towards their diagnosis. Psychological, physical, and spiritual support, including counseling, strengthened patients’ resilience and hope [14]. Family support [25] and spiritual beliefs also played key roles in helping patients accept their condition and feel more at peace with their diagnosis [25,26]. Care received by some patients also elicited hope and courage towards sustained treatment [11]. This was reiterated in the study by Harding et al. 2014 [21], who noted that older adults had better physical, psychological, and spiritual existential factor scores, which made them exhibit better attitudes noted through the ability to easily discuss their feelings and engage in planning treatment discussions.

#### Barriers to palliative care

The barriers to accessing palliative care were identified in 15 studies and synthesized into three main themes including limited knowledge and awareness, inadequate finances and systemic barriers.

##### Limited knowledge and awareness

In 10 studies [12,13,15,16,18,19,21–23,25], the lack of knowledge [12,13,16,18,22,25] and limited awareness [18,19] was highlighted as a significant barrier. This encompassed inadequate understanding of palliative care services [23], limited information on how to access care [15,19,21], cultural misconceptions [12,16], or stigma [12,13,23] surrounding cancer and palliative care, which often lead to late referrals at hospitals [12,16].

##### Inadequate finances

Seven studies [11–13,16,19,24,25] reported financial barriers, such as poverty [11,25], socioeconomic problems [13], insufficient funding [17,22] and lack of financial support [19]. Patients often could not afford outpatient services [19], leading to delayed care [16] or lack of access to necessary treatments.

##### Systemic barriers.

Again, seven studies [11–14,17,20,23] pointed to systemic issues, including a shortage of trained healthcare professionals [12], limited facilities [17], long wait times for diagnosis and treatment [13], and the absence of medical supplies [23]. Other factors, such as long travel distances [14], overcrowded hospitals [14], long queues [11,20], and dissatisfaction with payment systems [23], further hinder access to palliative care.

#### Adult cancer patients needs/expectations on accessing and receiving palliative care in SSA

The needs and expectations of adult cancer patients were synthesized into three themes: psychological and spiritual, socioeconomic, and physical.

##### Psychological and spiritual needs

Of the selected studies, 10 [11,14–16,18–20,22,24,26] reported a strong need for psychological [14,15,18,26] and spiritual support [16,19], including emotional care [22], religious guidance [26], and alleviation of distressing worry [20], depression, and stress [24]. Older patients often emphasized spiritual needs, while younger patients focused more on psychological support [19]. Patients sought care systems that addressed both emotional and spiritual well-being and supported them with more hope, faith, and courage [11].

##### Socioeconomic needs

Half of the selected studies [13–15,20–23,25] identified socioeconomic needs such as the expectation for social support [14,21,23], better access to care information [13,15,21–23] and improved healthcare services [13]. Patients also expressed the need for financial assistance [15,23], reduced hunger [20], more empathetic and confidential communication [23] and referrals to specialized healthcare facilities [25].

##### Physical needs

Four studies [15,18,20,21] highlighted the need for better pain control and symptom management with many patients not receiving sufficient relief from pain [18] despite the demand for effective medication and care.

A summary of the findings regarding the benefits, barriers, and expectations of adult cancer patients receiving palliative care in SSA is presented in Tables 3 and 4.

**Table 3.** A summary of key quantitative findings for the benefits, barriers, and expectations of adult cancer patients receiving palliative care in SSA.

Authors Country Year Article no.	Benefits of Palliative Care	Barriers/Challenges to Accessing & Receiving Care	Patient’s Needs/Expectations
Akuoko et al. 2022 Ghana [19]	–	The inability of most patients to access outpatient services due to financial incapability (96.6%), as well as limited awareness and limited information about palliative care	More supportive care and high spiritual needs (92.1%). Most older women (51.7%) reported more religious needs, and younger women (48.3%) reported more significant psychological and less religious needs.
Lazenby et al. 2016 Botswana [20]	–	Patient’s pain and symptoms, as well as worry, fatigue, hunger, long queues, and low functional dependencies, may contribute to patient’s inability to access care	The need to alleviate distressing worry, enhance symptom management and reduce hunger among patients.
Omoyeni et al. 2014 Nigeria [12]	Relief of distressing pain, satisfaction with care and the construction of the pain relief plan. Subsidised funds	Inadequate knowledge (fear of stigmatisation induced by culture), late presentation at hospitals	–
Olaitan et al. 2016 Nigeria [16]	Reduced suffering and improved quality of patient’s life	Late referral and presentation of patients to hospital due to cultural barriers, financial constraints as well as inadequate knowledge in prescribing palliative care.	Need to tackle psychosocial and spiritual issues
Tarus et al 2022 Kenya [24]	Few patients (27%) felt relief and were able to return home after management of their pain and breast wounds	Inadequate fund coverage for patient care	Need for psychological and spiritual support, as approximately half of the patients voiced worry, depression, and stress, with a small percentage (<10%) of them voicing spiritual distress.
Harding et al. 2014 Kenya and Uganda [21]	Some patients experienced better physical/psychological and existential/spiritual well-being with increasing age.	Inadequate information to navigate palliative care	Need for more pain control, social support and better access to information necessary to plan
Amare et al. 2023 Ethiopia [17]	–	Inadequate facilities & and services and a shortage of trained healthcare providers in the institution	More palliative care requirement is needed in males than females as patient age increases.
Ndiok et al. 2018 South Africa [15]	A helpful guide to developing a model for the integration of palliative care activities for patients was attained	Inadequate information on possibilities of treatment and side effects	Provision of Information, symptom care needs, psychological, spiritual and financial needs
Dlamini et al. 2020 South Africa [13]	–	Lack of cancer knowledge (stigma and discrimination), long waiting periods for referral, diagnosis, treatment and care, lack of screening and diagnostic equipment and socioeconomic barriers were challenges reported in the study.	Need for more cancer awareness campaigns, improved healthcare services and continuous professional development for healthcare providers

**Table 4.** A summary of key qualitative findings for the benefits, barriers, and expectations of adult cancer patients receiving palliative care in SSA.

Authors Country Year Article no.	Benefits of Palliative Care	Barriers/Challenges to Accessing & Receiving Care	Patient’s Needs/Expectations
Kebebew et al. 2022 Ethiopia [18]	Access to anti-pain treatment and support in cash or kind	Inadequate knowledge and low awareness regarding services rendered in palliative care.	More support. Efficient pain and symptom management, as well as psychological support during care. Only about two-thirds of patients (56.3% out of 90.3%) had been ‘fairly’ or ‘completely’ relieved of pain.
Lambert, et al. 2020 South Africa [14]	Uplifted survivors’ spirit, renewed hope due to support felt and increased will to live. Low-cost solution for health systems and improved survival rates among cancer patients	Stress, distance and cost of transportation to overcrowded clinics/hospitals were generally stated as barriers	More psychological and social support from care providers, family, religious and cancer peer groups
Natuhwera et al. 2021 South-Western Uganda [25]	Patients felt supported and positive because their needs were considered. They experienced improved family relationships and adaptation to accept the reality of the cancer diagnosis and disease progression	Poverty and a lack of knowledge observed in some patients	Expectations of patients to be referred to tertiary healthcare facilities (including cancer treatment centres) and to have more access to screening services scaled up to community-level healthcare facilities.
Nkoana et al. 2022 South Africa [26]	Important implications for the quality of life in survivors. Religious support helped survivors cope and deal with the life-threatening disease	–	The desire for systems of care that attend to the psychological and religious needs of patients
Namukwaya et al. 2022 Uganda, Nigeria and Zimbabwe [23]	Improvements in the management of patient’s symptoms. Quick service response in terms of distress. Satisfaction with care support. Renewed hope in life and reduced participants’ fear of their illness	Limited understanding/stigmatisation, Lack of continuity in consulting same health care professional. Unavailability of frequent medical supplies and medications. Frustration with being asked to pay for consultation prior to seeing health care professionals.	Patients expected communication to be empathetic and confidential, and they sought a diverse array of services, especially continuity of being attended to by the same health professionals, increased information, financial, social and psychological support
Chona et al. 2023 Tanzania [22]	–	Inadequate knowledge (about treatment modalities) indecision to undergo treatment/care as it is believed to be a waste of resources.	Need for psychological and emotional support, socioeconomic support and sensitisation or awareness generally to enlighten spouses, family and community members
Anarado et al. 2017 Nigeria [11]	Some patients reported that they got care that elicited hope, faith and courage, which led them to have sustained treatment	The inability of patients to accept and navigate through treatments and financial demands	Supporting patients with more hope, faith and courage for sustained treatment; self-care actions

## Discussion

This study systematically reviewed recent literature on access to palliative care services for adult cancer patients in sub-Saharan Africa (SSA). It focuses on the benefits, barriers, and expectations from the patients’ perspective. The findings reveal that palliative care is often out of reach for many cancer patients in SSA, particularly older adults, and is underutilized [23,27]. The benefits identified in this study were improved patient satisfaction, quality of life, and a better attitude toward diagnosis. Key barriers include limited knowledge and limited awareness, inadequate finances, and systemic barriers. Patients’ expectations highlighted the need for more comprehensive care, including socioeconomic, spiritual, psychosocial, and physical support.

This study found that palliative care was reported in only nine out of approximately 48 sub-Saharan African (SSA) countries, with many countries either underrepresented or not represented. The lack of widespread palliative care coverage is attributed mainly to the region’s insufficient human and financial resources [28]. The research also identified a higher number of female cancer patients in the studies, likely due to the higher incidence of gynecological cancers in women, as reported by the WHO [5]. Additionally, the study found that older patients generally require more palliative care due to multiple chronic conditions [3,17]. Interestingly, more men were reported to need palliative care than women, which may be linked to the higher prevalence of aggressive cancers in men [17].

The studies reviewed highlight the diverse modes of palliative care provided to adult cancer patients, including hospital-based, home-based, home visit, phone-based, and mobile hospice services. Public health systems, private clinics, NGOs, and national cancer treatment centers supported these services. Palliative care generally encompassed physical, social, psychological, spiritual, and mental support delivered by healthcare professionals, family, religious groups, and counsellors [12,14,16,18,24]. While the studies did not specify the factors influencing the choice of palliative care model, it can be inferred that decisions were shaped by the organizational structure, available resources, and the expertise of the healthcare team, as well as the inclusion of culturally appropriate care [23,29]. The World Health Organization advocates for a comprehensive, person-centred approach [5]. A key recommendation emerging from the studies is the early introduction of palliative care alongside curative treatment to maximize benefits for cancer patients [23,30].

In sub-Saharan Africa (SSA), while palliative care for adult cancer patients has been introduced, its early stages and its benefits are still not comparable to those seen in high-income countries with more developed health settings [28]. Despite establishing the first hospice in Zimbabwe and ongoing efforts to expand palliative care services in SSA, it remains underdeveloped [1,31] due to limited integration into mainstream public health systems, regional differences, and the rising burden of non-communicable diseases [31]. Approximately 68% of the studies selected for this review highlighted the benefits of palliative care and have been synthesized into main categories of improved quality of life, patient satisfaction, and a better attitude towards diagnosis. The current review highlighted that patient access to palliative care was associated with improved quality of life, particularly through reduced symptom burden, better pain, and wound management, and advanced care planning [16,18]. Additionally, palliative care patients reported better social, spiritual, and psychological well-being, enhancing their quality of life [21,26]. Spirituality and religiosity, in particular, were linked to a higher quality of life among cancer patients in developing countries [32].

Palliative care was reported to facilitate better communication, enabling patients to set realistic expectations and achieve personal goals, while providing social support [33]. In particular, combining palliative care with geriatric medicine in multidisciplinary teams has been shown to improve quality of life among older cancer patients, helping them manage symptoms more effectively [34]. When patients had the opportunity to discuss their feelings and engage in planning their care, they reported better physical, psychological, and existential well-being [21]. This proves that access to multidisciplinary palliative care services may improve the attitude of patients towards diagnosis as well as patient satisfaction.

A recent study in Sweden demonstrated that integrating a structured palliative care guide into geriatrics significantly improved patient satisfaction, with higher scores in nine out of 10 questions (*p* = 0.02 to < 0.001) in the intervention group compared to baseline, each group consisting of 200 patients [35]. This reiterates that a well-organized, multidisciplinary approach to palliative care can lead to enhanced outcomes, including increased patient satisfaction.

Building on these findings, it can be inferred that adopting a similar structured palliative care model in sub-Saharan Africa (SSA) could offer substantial benefits, such as reducing symptom burden, improving patient satisfaction, and potentially extending life expectancy. Given these results, implementing a well-defined care guide for adult cancer patients in SSA may lead to similar positive outcomes, including better symptom management and prolonged life [36].

In low- and middle-income countries, particularly in sub-Saharan Africa (SSA), palliative care faces significant challenges. In the current literature review, about 93% of studies identified barriers, synthesized into three main categories: lack of knowledge and awareness, inadequate financial resources, and systemic barriers. Many patients are unaware of available outpatient services [19], and some do not understand how palliative care alleviates pain [18]. Some even hesitate to pursue care, viewing it as a waste of resources [22]. These barriers persist due to limited education, resource scarcity, and long hospital delays, geographic access problems, and shortages of qualified healthcare workers.

Cultural misconceptions further exacerbate these challenges. In some communities, cancer is viewed as contagious, akin to human immunodeficiency virus (HIV) [23], while others associate the disease with supernatural forces or bad luck [22,26]. These cultural beliefs contribute to late-stage hospital presentations [16,26] and reluctance to explore access to care options [12,13] as patients fear stigmatization or discrimination. Socioeconomic poverty and a lack of trained healthcare professionals are additional barriers, creating a vicious cycle of inadequate care [28,31,37]. These findings are consistent with previous studies highlighting cultural misunderstandings and sensitivity, resource scarcity, and weak health policies as significant obstacles to effective palliative care in SSA [7,28,31].

Addressing these barriers could benefit from community engagement efforts, similar to those seen in some developing countries, which help to raise awareness and reduce stigma [38]. In addition, incorporating advanced care planning (ACP), a communication approach encouraging patients to document their preferences for care has improved palliative care outcomes in high-income countries [34]. Implementing advanced care planning in SSA could help patients better navigate available care services while also considering cultural sensitivity and ensuring that their needs are met in a timely and personalized manner.

Adequate resource allocation is also critical for improving palliative care in SSA [36]. High-income countries benefit from well-organized healthcare systems that provide comprehensive cancer care [39], while low- and middle-income countries face much higher health burdens with fewer resources [13,20,27]. If SSA countries can allocate more funds to cancer treatment and palliative care services, many logistical challenges, such as transportation and proximity to care centers, could be addressed [40]. This could be achieved through better financial management, including organized healthcare budgets, mass health insurance schemes, and subsidies for patient care.

Finally, the successful integration of palliative care into cancer treatment in SSA will require strong leadership and policy reforms [36]. A study from Uganda emphasized that effective healthcare policies and robust management are essential for overcoming systemic challenges and improving care outcomes [2]. Strengthening leadership, investing in healthcare infrastructure, training skilled professionals, and designing more coordinated palliative care programs tailored to SSA’s needs will significantly improve the region’s access to palliative care for adult cancer patients [2,7,40].

Adult cancer patients in sub-Saharan Africa (SSA) have expressed key expectations for palliative care, categorized into socioeconomic, psychosocial, spiritual, and physical needs. As synthesized in this study, socioeconomic support encompasses the patients’ need to access efficient medical care [13], clear information about palliative care options [21,22], and basic needs like food [20]. Psychosocial and spiritual support includes the need for meaning and hope [26], self-worth, coping abilities [11], and spiritual care to address distress and ensure patients feel valued. A similar review that discusses palliative cancer care needs in SSA reported that medication-based pain relief is minimal and, therefore, highly needed in low-income countries compared to high-income countries [41]. This reiterates the need for pain control and symptom management, which are highlighted as physical needs in this study [18,20,21]. However, these needs are unlikely to be fully met in SSA without improved policies and resource allocation.

Adopting a structured palliative care model, such as the one used in Sweden, could offer significant benefits for improving the quality of palliative care in sub-Saharan Africa (SSA). However, substantial adaptation to local cultural, socio-economic, and healthcare contexts is required for such a model to be effective in SSA. Sweden’s patient-centered, multidisciplinary approach to palliative care, which focuses on comprehensive symptom management, advance care planning (ACP), and holistic care, could help address the growing need for palliative services in SSA, particularly for patients with life-limiting diseases like cancer [34,35].

Yet, implementing this model in SSA would face several key challenges, such as cultural differences prioritizing family-centered decision-making over individual autonomy, resource limitations, and healthcare infrastructure deficits [32,40]. These challenges underscore the need for a flexible and contextually sensitive approach that adapts Sweden’s model to the realities of SSA, where healthcare systems are often strained by a high burden of infectious diseases, limited funding, and inadequately trained professionals [2,7,31].

The model must prioritize community-based care to overcome these barriers, emphasizing training healthcare workers and incorporating culturally sensitive ACP practices [29]. Importantly, the model’s success in SSA would depend on implementing pilot programs, international partnerships, and scalable, low-cost solutions that can be easily adapted to local resources [1,41,42]. With the proper adjustments, such a structured model could significantly expand access to palliative care, improve patient outcomes, and effectively address chronic diseases prevalent in SSA.

The global impact of enhancing palliative care services in SSA extends far beyond the region, influencing worldwide health policies and practices. As cancer rates continue to rise, particularly in low-resource settings, the lessons learned from SSA could provide valuable insights for other regions facing similar challenges in delivering comprehensive healthcare. Integrating palliative care into healthcare systems in SSA can serve as a model for improving care in underserved regions globally. As outlined by the World Health Organization [43], people-centered care that addresses not just the physical but also the psychological and spiritual needs of patients are key to achieving universal health coverage. This approach has significant implications for global health, as it aligns with the WHO’s health goals and directly addresses the broader burden of rapidly increasing non-communicable diseases like cancer [4]. In SSA, the projected rise in cancer incidence demands urgent action to improve care, with palliative services playing a crucial role in alleviating suffering and enhancing the quality of life for patients [44]. By addressing systemic challenges such as financial barriers, inadequate awareness, and socio-political issues, SSA’s experience provides a pivotal opportunity for learning and improvement that can guide global efforts to strengthen the healthcare system and provide equitable care. Ultimately, improving palliative care in SSA benefits local populations and contributes to the global conversation about effective, accessible, compassionate care for all.

This study recommends several key actions to improve access to palliative care for adult cancer patients in sub-Saharan Africa (SSA). These include raising awareness about palliative care, engaging communities, ensuring high standards of care, investing in healthcare practitioner training, and developing comprehensive training programs. Strengthening healthcare infrastructure, integrating palliative care at all levels, and promoting interdisciplinary care models and timely access are also crucial. Additionally, conducting palliative care research and standardizing care across regional health guidelines are necessary [2,34,36,37,41]. However, successful implementation requires considering the population’s cultural, geographical, and socioeconomic contexts [29].

This research acknowledges several limitations that could affect its findings. First, publication bias may have occurred due to language restrictions and the exclusion of theses and conference papers, potentially missing valuable data. Additionally, the study was limited to just 9 out of 49 sub-Saharan African (SSA) countries, which may reduce the generalizability of the results. Future studies could include more diverse regions by translating papers and collaborating with local researchers. Another limitation is the lack of research on palliative care for older adults in SSA, which may have led to reporting bias, especially regarding older cancer patients’ experiences with palliative care. Despite these limitations, the study has notable strengths, including a registered protocol ensuring transparency and using the rigorous JBI method to assess evidence quality. The findings highlight the importance of palliative care for cancer patients in SSA and could inform policies to improve palliative care practices, especially for older adults.

## Conclusion

In conclusion, while palliative care is available to adult cancer patients in sub-Saharan Africa (SSA), its effectiveness is hindered by barriers like lack of awareness, financial constraints, and socio-political challenges. Many patients, especially those with advanced cancer, experience unmet physical, psychosocial, and spiritual needs. Raising awareness, improving education, and creating policies to fund palliative care are crucial to address this. Integrating palliative care into cancer control strategies and national health policies is also key. Effective change will require coordinated efforts from healthcare providers, policymakers, and communities. Investing in training, public education, and strengthening health systems will help overcome these barriers and ensure better care for cancer patients in SSA.

## Supplementary Material

PRISMA checklist.doc

## Data Availability

The data used during the current study are available from the corresponding author upon reasonable request.
